# Emotional Intelligence and Emotional Hypersensitivity in Gifted Individuals

**DOI:** 10.3390/jintelligence11020020

**Published:** 2023-01-17

**Authors:** Christelle Gillioz, Maroussia Nicolet-dit-Félix, Marina Fiori

**Affiliations:** Research & Development, Swiss Federal University for Vocational Education and Training (SFUVET), 1020 Renens, Switzerland

**Keywords:** emotion–information processing, emotional intelligence, high IQ, Mensa members, hypersensitivity

## Abstract

The goal of the present study was to investigate the associations between high intelligence, emotional intelligence (EI), and emotional hypersensitivity in a sample of 304 Mensa members. In addition, we aimed to shed light on how highly intelligent individuals process emotional information. In a previous study, we found that individuals with high EI in the general population are characterized by an attentional bias toward emotional information. We tested whether this effect holds for highly intelligent individuals by drawing on the same procedure: participants (N = 124 Mensa members) had to report a letter appearing behind a picture of a face with emotional or a neutral facial expression, and their reaction time to provide an answer was recorded. Comparing the results from the general population to those of Mensa members, we found that Mensa members did not show the attentional bias toward emotional information found in the general population. Mensa members were equally fast to evaluate letters replacing emotional and neutral expressions, and this result was not influenced by EI level. Possible explanations include the role of inhibitory processes (a factor related to intelligence), which might have contributed to treating emotional information as purely cognitive.

## 1. Introduction

Common views of individuals with intellectual giftedness (hereafter referred to as giftedness) often portray them as stereotypes, such as troubled geniuses (e.g., Will Hunting) or nerds with no social life (e.g., Sheldon Cooper from the Big Bang Theory). The underlying idea is that giftedness not only relates to higher intellectual capacities but should also manifest itself in deficiencies in other domains, such as socio-emotional skills, well-being, or other personal characteristics. This view that giftedness comes at a cost and must be accompanied by difficulties is consistent with the *disharmony hypothesis* (which stems from the “mad genius” myth (Becker 1978)) and is prevalent in mass media (Bergold et al. 2021), laypersons’ representations (Baudson 2016) or teachers’ and psychologists’ conceptions (Matheis et al. 2020; Sanchez et al. 2022).

In contrast, the *harmony hypothesis* (e.g., Persson 1998) states that gifted individuals are socially and emotionally equal or superior to their non-gifted counterparts. In this view, gifted people are not only more intelligent, but they are also “more” in other domains and can better adjust and more easily adapt to new circumstances.

Empirical findings do not support the disharmony hypothesis, with studies showing that gifted participants do not differ from non-gifted participants on characteristics such as mental disorders, motivation, subjective well-being, life satisfaction, or satisfaction with relationships (Martin et al. 2010; Rinn and Bishop 2015). When, in fact, differences were found between gifted and non-gifted individuals, they tended to support the harmony hypothesis by showing an advantage for the gifted group. For example, gifted adolescents were perceived by their peers as less aggressive and more prosocial (Peairs et al. 2019) and were more popular than their non-gifted classmates (Czeschlik and Rost 1995).

Another stereotype commonly associated with giftedness is hypersensitivity. A web search for characteristics of giftedness rapidly reveals that high levels of intensity or hypersensitivity are almost always mentioned as part of the giftedness package. Again, there is low empirical evidence on this question, and the findings differ depending on what is referred to as hypersensitivity.

Hypersensitivity is a recent and rather broad concept that corresponds to heightened sensory (visual, auditive, olfactive, etc.) and emotional sensitivity. As defined by Aron and Aron (1997), hypersensitivity is an innate difference called *sensory-processing sensitivity*, which can be assessed with a questionnaire, the Highly Sensitive Person Scale (HSPS), evaluating perceptions of sensory stimulation, emotions, associated personality traits, and behaviors. Based on this questionnaire, 15–20 percent of the population are hypersensitive, meaning that they are more sensitive to sensory stimulation and react in a stronger way. Regarding the relationship between giftedness and hypersensitivity, some studies have suggested that sensory sensitivity increases with IQ (Deary et al. 2004; Schlegel et al. 2017). Yet, these findings, while showing an association between intelligence and sensitivity, are not sufficient to draw conclusions on hypersensitivity and giftedness.

Another way to conceptualize hypersensitivity is to turn to the theory of positive disintegration (TPD, Dabrowski 1964) and the concept of overexcitability (OE) that corresponds to a higher response to stimuli. OEs can be related to one or all of five domains: psychomotor, sensual, emotional, imaginational, and intellectual. Different researchers have claimed that gifted individuals are more overexcitable than the general population (e.g., Piechowski 1997) and that OEs could be used to detect giftedness. A recent meta-analysis (Winkler and Voight 2016) provided clarity about the relationship between OE and giftedness, showing that, generally, gifted students have higher scores on all OE subdomains except the psychomotor one. Importantly, in terms of effect sizes, differences between gifted and non-gifted populations were small to medium, which questions the fact that gifted individuals are characterized by higher OEs.

Despite these findings, giftedness is still largely associated with stereotypes in our society, especially regarding emotional competence. Gifted people are seen as having a qualitatively different way of processing and managing emotions (Brasseur 2021). For example, gifted students are described as more introverted, less emotionally stable, and less agreeable (Baudson and Preckel 2013).

One way to understand the association between high IQ and emotional competencies is by employing the construct of emotional intelligence.

### 1.1. Relationship between Emotional Intelligence and IQ

Emotional intelligence (EI) concerns the abilities and self-perceptions related to the recognition, expression, understanding, and management of emotions. There are two major conceptualizations of EI. The first, trait EI, defines EI as a personality trait, which can be measured with a self-report questionnaire. People respond to questions about emotions as “I usually find it difficult to regulate my emotions,” to calculate their trait EI score. The second conceptualization, ability EI, considers EI an ability (i.e., as a form of intelligence) that can be measured with performance tests, each addressing a different facet of EI: emotion perception, emotion understanding, and emotion management. For example, the emotion understanding facet of EI is measured with short scenarios in which the task is to identify the associated emotion. Different from trait EI questionnaires, which are self-perceptions reflecting how people behave in their daily life (or their typical performance), ability EI tests are based on correct answers and assess how individuals perform tasks that require concentration and effort (or maximum performance). In order to test the association between high IQ and EI, in the current investigation, we measured both trait and ability EI.

Ability EI shows low to moderate correlations with intelligence, a finding that overall supports the conceptualization of EI as a form of intelligence. In addition, a seminal work on the relationship between intelligence and EI modeled EI and the three ability EI components of perception, understanding, and management of emotions as a second stratum factor of the Cattell-Horn-Carroll model of cognitive ability (MacCann et al. 2014).

According to the “positive manifold” idea, which proposes that the score obtained by an individual on one mental ability predicts scores on another, if individuals are high on intelligence, they should also be high on EI.

Scientific literature showed that when EI is considered a trait, the link between intelligence and EI is rather inconsistent, with studies showing that gifted students score higher on trait EI (e.g., Lupu 2012) compared to their non-gifted counterparts and studies showing opposite (e.g., Dijkstra et al. 2012; Zeidner et al. 2005) or null results (Angela and Caterina 2022).

When EI is considered an ability, results become more coherent; namely, EI is positively associated with intelligence (e.g., Castro-Johnson and Wang 2003; Egeland 2019; Valadez Sierra et al. 2013; Zeidner et al. 2005). A recent meta-analysis revealed that gifted individuals scored slightly higher on EI, but only when ability EI was considered (Ogurlu 2021). All in all, it seems that the association between EI and intelligence depends on the approach employed to conceptualize EI (trait vs. ability) and on the specific measures used, especially for findings related to trait EI.

### 1.2. Recent Theorizations about EI and Intelligence

Among the most significant challenges in the literature on EI has been the introduction of tests that measure EI as an ability—that is, as a form of intelligence. In-depth analysis of the typical items employed to measure EI as an ability reveals that individuals may correctly answer test questions by relying on what they know about emotions. This begs the question as to whether they would be able to apply that knowledge in real-life situations. A recent approach to EI introduces a new component that measures how individuals experience (feel) and respond to emotions. Such a component represents “experiential” EI and is deemed to predict additional variability in emotionally intelligent behavior. More specifically, this new theorization posits that within a broad conceptualization of EI as a unique construct, there might be two distinct components: (1) EI_K_ or the emotional knowledge component, measured with current ability emotional intelligence tests, and related to higher-order reasoning, or top-down processing, about emotions; (2) EI_P_ or the emotional information processing component, measured with emotional information processing tasks and based on attention-related responses to the emotional information, or bottom-up processing (Fiori et al. 2022). EI_K_ shares similar characteristics with crystallized intelligence (Gc) as it refers to a reflexive ability based on prior acquired knowledge and assessed through maximum performance tests, while EI_P_ shares similar peculiarities with fluid intelligence (Gf) as it describes more automatic and fast processing of information and can be measured with cognitive tasks, such as the emotional Stroop task or the emotional GoNogo task.

In a previous study conducted in the general population testing the association between EI_K_ and EI_P_ (Nicolet-dit-Félix et al. 2023), it was found that people high in the emotion understanding facet of EI (EI_K_) preferably directed their attention toward emotional stimuli, as reflected in the difference in reaction time toward letters replacing emotional versus neutral facial expressions in a dot-probe task (EI_P_). In particular, at levels of emotional understanding greater than one standard deviation from the mean score, the difference between attention to emotional and neutral expressions became evident.

In principle, because of the positive relationship between IQ and ability EI, gifted (and, therefore, with high EI_K_) individuals should show the same preference for emotional vs. neutral information as the one found in the general population. However, the fact that gifted individuals are generally higher on EI_K_ does not automatically entail that such individuals would be higher on EI_P_ too. For example, gifted individuals might be characterized by wider emotional knowledge, but no specific attentional bias toward emotional information. One of the goals of this study was to shed light on this question by replicating the study from Nicolet-dit-Félix et al. (2023) and comparing the results found in Mensa members to those of non-gifted individuals.

### 1.3. The Current Research

The aim of the current study was twofold. First, we aimed to collect descriptive information regarding the characteristics of gifted adults. To this purpose, we included measures of demographic characteristics, the prevalence of mental disorders, different aspects of personality, and variables commonly associated with giftedness, such as hypersensitivity, satisfaction with life, or quality of interpersonal relationships and ability and trait EI. We assessed how gifted individuals performed on the three facets of ability EI and how they evaluated their trait EI. We also collected information regarding perceived stress, satisfaction with life, and the quality of interpersonal relationships. Second, we aimed to test whether gifted individuals showed attentional bias toward emotional stimuli similar to what was found in the general population (Nicolet-dit-Félix et al. 2023). To do so, we replicated Nicolet-dit-Félix et al.’s study on a sample of gifted individuals and employed the dot-probe (DP) paradigm to measure EI_P_. In this task, participants had to identify and report a target letter appearing behind one of two faces appearing for a very limited time (i.e., 100 ms) on the screen. One of the two faces expressed anger or happiness, and the other was emotionally neutral. Attentional bias toward emotional information was operationalized as faster reaction times in the emotional condition (i.e., when the probe appeared at the location of the emotional face) compared to the neutral condition (i.e., when the probe appeared at the location of the neutral face), reflecting the fact that the participant’s gaze was attracted by the emotional facial expression.

If high-EI gifted individuals are characterized by a preference for emotional information, they should be more “caught” by emotional stimuli compared to neutral ones. This attraction should involve faster reaction times when judging the target letter replacing an emotional as compared to a neutral face. In this case, we would expect the difference in reaction times between the neutral and emotional conditions to increase with EI, in line with what was found in the general population. In contrast, if gifted individuals are only high in EI_K_ but not in EI_P_, their reaction times should not differ between the neutral and emotional conditions, even at high levels of EI.

As gifted adults are hard to find in the population, we recruited them through the Mensa society. This organization includes people belonging to the most intelligent two percent of the population. Mensa members have an IQ above 130^1^ on an approved intelligence test. Today, there are around 145,000 Mensa members in roughly 90 countries throughout the world (Mensa International, n.d.).

## 2. Method

### 2.1. Participants and Procedure

The sample included 327 Mensa members (English-speaking = 266, French-speaking = 61) recruited through Mensa Switzerland and Mensa UK. Participants took part in the first session, in which they completed a battery of questionnaires assessing their emotional intelligence and other characteristics. As the first session lasted about an hour and was completed online, we excluded participants who did not pass the attentional check or who had a mean below 0.1 on the emotion recognition test (corresponding to less than 4 correct items out of 42 items). The sample of the first session consisted of 304 Mensa members (English-speaking = 247, French-speaking = 57; 150 male, 153 female, 1 other). The Mensa sample was quite varied, with participants aged from 18 to 81 years old, the level of highest completed education ranging from compulsory education to doctoral degree, and from different living areas (Table 1).

After the first session, participants were asked to take part in the second session, consisting of the DP task. Trait EI was also evaluated at this time. In this session, 125 Mensa members (58 male, 66 female, and 1 other) completed both sessions. All participants were informed about the study and gave their consent to participate following procedures and protocols approved by the ethical committee of the University of Geneva.

As a control group, we included non-gifted participants from Nicolet-dit-Félix et al. (2023), recruited on Prolific. The control sample involved 157 individuals (52 male, 103 female, and 2 who indicated “other”), aged between 18 and 63 (*M* = 28.9, *SD* = 9.8).

### 2.2. Measures

#### 2.2.1. The Situational Test of Emotional Understanding-Brief (STEU-SF, MacCann and Roberts 2008) and The Situational Test of Emotional Understanding-Brief (STEU-B, Allen et al. 2014)

The STEU-SF and the STEU-B are the performance-based tests that evaluate the understanding of emotions felt by protagonists pictured in short scenarios such as in “Xavier completes a difficult task on time and under budget. Xavier is most likely to feel?” (Pride). Responses are scored as correct (1) and or incorrect (0). The STEU-SF comprises 25 items, and the STEU-B 19 items. The test–rest reliability of the full version of the test is .72 (Libbrecht and Lievens 2012). Mensa participants were evaluated with the STEU-SF and non-gifted individuals with the STEU-B.

#### 2.2.2. The Situational Test of Emotional Management-Brief (STEM-SF, MacCann and Roberts 2008) and the Situational Test of Emotional Management-Brief (STEM-B, Allen et al. 2015)

The STEM-SF and the STEM-B are performance-based tests that evaluate the knowledge of the way to manage emotions in various situations. Responses are scored according to a weight derived from expert ratings. For instance, for the item “Wai-Hin and Connie have shared an office for years, but Wai-Hin gets a new job, and Connie loses contact with her. What action would be the most effective for Connie?”, the most appropriate response is “Contact Wai-Hin and arrange to catch up but also make friends with her replacement.” The STEM-SF comprises 20 items, and the STEM-B 18 items. The test–rest reliability of the full version of the test is .85 (Libbrecht and Lievens 2012). Mensa participants were evaluated with the STEM-SF and non-gifted individuals with the STEM-B.

#### 2.2.3. The Geneva Emotion Recognition Test Short Version (GERT-S, Schlegel and Scherer 2016)

The GERT-S is a 42-item performance-based test that evaluates the ability to recognize emotions in the face, voice, and body. During the test, 42 short video clips with sound (duration 1–3 s), in which professional actors express 14 different emotions, are presented, and respondents have to select which emotion is represented in the video. The responses are scored as correct or incorrect. The Cronbach alpha was .63 in our sample.

#### 2.2.4. Trait Emotional Intelligence Questionnaire Short Form (TEIQue-SF, Version 1.50, Cooper and Petrides 2010)

The TEIQue-SF is a 30-item inventory that measures global trait emotional intelligence. Respondents are asked to rate on a Likert scale, ranging from 1 = *Completely Disagree* to 7 = *Completely Agree*, their degree of agreement with different statements, such as “Expressing my emotions with words is not a problem for me.” The global trait EI score is calculated by summing the item scores and dividing by the total number of items. The Cronbach alpha was .89 in our sample.

#### 2.2.5. The Affect Intensity Measure Short Form (AIM-SF, Larsen 1984)

The AIM-SF is a 20-item questionnaire that evaluates how respondents react to typical life events, with items such as “I get upset easily”. Respondents are asked to rate on a Likert scale, ranging from 1 = *Never* to 6 = *Almost always*, how often they react in a described way. The Cronbach alpha was .83 in our sample.

#### 2.2.6. Positive and Negative Affect Schedule (PANAS-SF, Watson et al. 1988)

The PANAS-SF is composed of two 10-item mood scales assessing positive and negative affect. Respondents are asked to indicate on a Likert scale, ranging from 1 = *Very slightly or not at all* to 5 = *Extremely*, the extent to which they have felt a particular affect over the past week. Positive Affect (PA) correspond to *interested, excited, strong, enthusiastic, proud, alert, inspired, determined, attentive,* and *active*, while Negative Affect (NA) correspond to *distressed, upset, guilty, scared, hostile, irritable, ashamed, nervous, jittery,* and *afraid*. The Cronbach alpha was .88 for PA and .87 for NA.

#### 2.2.7. The Perth Emotional Reactivity Scale Short Form (PERS-SF, Preece et al. 2019)

The PERS-SF is an 18-item inventory that evaluates reactivity to positive and negative emotions on three dimensions: activation, intensity, and duration. Examples of items include “I react to good news very quickly”. Respondents are asked to rate on a Likert scale, ranging from 1 = *Very unlike me* to 6 = *Very like me*, how they typically react to emotional events. The Cronbach alpha was .90 in our sample.

#### 2.2.8. The Highly Sensitive Person Scale (HSP-12, Pluess et al. 2020)

The HSP-12 scale is a 12-item short version of the original 27-item scale (Aron and Aron 1997) that examines environmental sensitivity. Respondents are asked to rate on a Likert scale, ranging from 1 = *Not at all* to 7 = *Extremely*, the extent to each item which applies to them. Examples of items include: “I am bothered by intense stimuli like loud noises or chaotic scenes” and “I make a point to avoid violent movies and TV shows”. The Cronbach alpha was .74 in our sample.

#### 2.2.9. The Perceived Stress Scale (PSS-10, Cohen and Williamson 1988)

The PSS-10 is a 10-item instrument measuring the perception of stress. Respondents are asked to indicate on a Likert scale, ranging from 0 = *Never* to 4 = *Very often*, the degree to which their life situations were appraised as stressful. Example of items includes: “In the last month, how often have you felt nervous and stressed?” The Cronbach alpha was .87 in our sample.

#### 2.2.10. The Satisfaction with Life Scale (SWLS, Diener et al. 1985)

The SWLS is a 5-item scale assessing global life satisfaction. Respondents are asked to indicate on a Likert scale, ranging from 1 = *Strongly disagree* to 7 = *Strongly agree*, their agreement with the statement related to their life satisfaction. Example of items includes: “The conditions of my life are excellent.” The Cronbach alpha was .87 in our sample.

#### 2.2.11. The Interpersonal Relationships Facet of Ryff Scale (Ryff 1989)

The interpersonal relationships facet of the Ryff scale is a 14-item scale. Respondents are asked to rate on a Likert scale, ranging from 1 = *Strongly disagree* to 7 = *Strongly agree*, the extent to which each item applies to them. Example of items includes: “I enjoy personal and mutual conversations with family members or friends”. The Cronbach alpha was .87 in our sample.

#### 2.2.12. Presence of Neurological or Psychological Disorders

Participants were asked to report whether they had symptoms or a medical diagnosis for the following disorders: anxiety, depression, bipolar disorder, borderline personality disorder, attention deficit hyperactivity disorder (ADHD), autism spectrum disorder (ASD), or Asperger’s syndrome. Participants could select several options. They could also add another disorder if necessary.

#### 2.2.13. Brief Mood Introspection Scale (BMIS, Mayer and Gaschke 1988)

Before starting the DP task, the participants were asked to evaluate their mood with the item “Overall, your mood right now is” from the BMIS. The response scale ranged from 0 = *Very unpleasant* to 10 = *Very pleasant*.

### 2.3. Dot-Probe (DP) Task

The same DP task as in Nicolet-dit-Félix et al. (2023) was used. In this task, participants had to identify and report a target letter appearing behind one of two faces presented for a limited time on the screen. One of the faces was emotional, and the other was neutral. Reaction times to identify the letter were recorded. The specific DP task used in this study is described hereafter.

#### 2.3.1. Material

A set of 16 angry, 16 happy, and 32 neutral-colored faces were selected from the Karolinska Directed Emotional Faces (Lundqvist et al. 1998). Each set was composed of an equal number of expressions displayed by female and male models with similar skin tones. The emotional faces were the most intense and arousing validated expressions of anger and happiness from series A (Goeleven et al. 2008, angry: *M*_int_ = 6.7, *SD*_int_ = 0.6, *M*_ar_ = 4.1, *SD*_ar_ = 0.6; happy: *M*_int_ = 6.9, *SD*_int_ = 0.3, *M*_ar_ = 4.1, *SD*_ar_ = 0.3; neutral: *M*_int_ = 4.7, *SD*_int_ = 0.4, *M*_ar_ = 2.6, *SD*_ar_ = 0.3). Using Adobe Photoshop Element 2021, all the stimuli were cropped into a standard oval shape concealing hair and external features and with the eyes aligned horizontally at the same height. The size of the stimuli was 346 × 460 pixels (approximately 12 × 16 cm).

#### 2.3.2. Procedure

**Figure 1:**
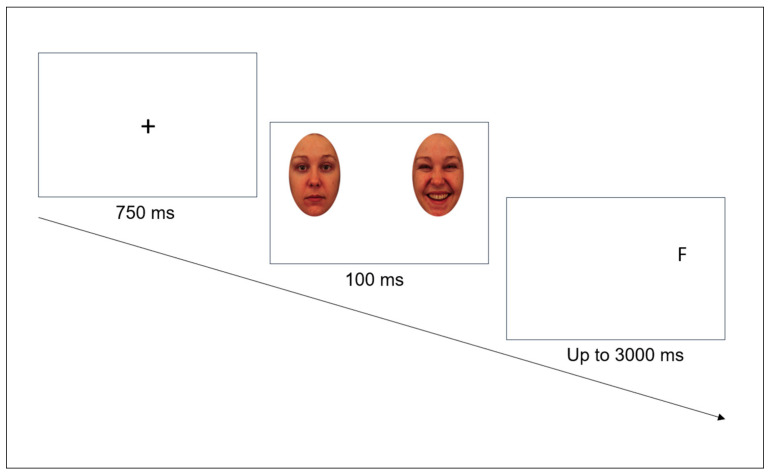
Example of a Trial in the Dot-Probe Task.

The task was adapted from the original version of the DP task (Posner 1980). It started with the presentation of a fixation cross at the center of the screen for 750 ms. Then, two faces were simultaneously displayed during 100 ms, one on the right and the other on the left side of the screen. One of the faces was emotional (anger or happiness), and the other one was neutral. After a 100 ms blank screen, a probe (i.e., the letter “F” or “H”) appeared with an equal chance at the location of the emotional face (emotional condition) or the neutral face (neutral condition). Participants had to report as quickly as possible the letter by pressing “F” or “H” on the keyboard. Letters disappeared after 3000 ms or less if the participant responded earlier (Figure 1). Reaction times to report the letter were recorded. The task started with 8 practice trials to allow participants to get familiarized with the task and to get feedback on their performance. Then, the main task was composed of 128 trials divided into 4 blocks of 32 trials. Each block contained 16 angry–neutral and 16 happy–neutral pairs expressed half the time by female and half the time by male models and presented in a randomized order. Three 30-s breaks between blocks were proposed to the participants to give them the opportunity to relax or to look again at the instructions. To ensure that participants stayed motivated throughout the task, we informed them that they would get feedback about their performance at the end of the task. The experiment was run online with the Gorilla interface (Anwyl-Irvine et al. 2020). We restricted access to Chrome and Edge browsers in order to maximize precision in presentation visual delay for all operating systems (Anwyl-Irvine et al. 2021).

## 3. Results

Hereafter, we first report descriptive information regarding the data on personal characteristics collected in the first wave of questionnaires before presenting the analyses related to the DP task.

### 3.1. Descriptive Statistics and Correlations between Variables

Mean, standard deviation, and Pearson correlations for all questionnaires of the first session for Mensa members (N = 303^2^) are shown in Table 2. For comparison purposes, this table also contains the means and standard deviations of the participants from Nicolet-dit-Félix et al. (2023). In the Mensa sample, age correlated positively with positive emotional reactivity (0.16) and negatively with emotion recognition (−0.31), affect intensity (−0.12), negative affect (−0.35), negative emotional reactivity (−0.19), sensory sensitivity (−0.17), and perceived stress (−0.22). Among ability EI measures, the understanding and management facets were positively correlated (0.29). Emotion understanding correlated negatively with affect intensity (−0.12) and sensory-processing sensitivity (−0.13). Emotion management correlated positively with positive emotional reactivity (0.14). Emotion recognition correlated positively with affect intensity (0.18), negative affect (0.17), sensory-processing sensitivity (0.24), and perceived stress (0.17), and negatively with positive emotional reactivity (−0.13). Affect intensity correlated positively with positive (0.24) and negative affect (0.28), emotional reactivity (positive: 0.29, negative: 0.38), sensory-processing sensitivity (0.47), and perceived stress (0.28).

Table 3 shows the percentages of participants who disclosed symptoms or diagnostics related to neurological or psychological disorders.

To investigate whether Mensa members differ from non-gifted individuals regarding their personality and variables commonly associated with giftedness, we compared their results to those obtained in a previous study on non-gifted participants, not reported in the corresponding article but available on demand (Nicolet-dit-Félix et al. 2023), regarding EI facets, affect intensity, positive and negative affect, satisfaction with life and perceived stress. For the remaining variables (i.e., reactivity to emotion and sensory-processing sensitivity), we relied on scores provided in recent studies. Because the Mensa sample was older and comprised more male participants than our control sample, we controlled for age and gender in the analyses. Since slightly different short-form tests were used to assess emotion understanding and emotion management in the Mensa and control samples, we verified that the difference between the groups did not come from the version employed by doing the analyses on means and on corrected means based on items present in both versions of the tests (14 items in each test).

Regarding EI, our sample had higher STEU scores (mean: *F*(1, 455) = 91.25, *p* < .001, corrected mean: *F*(1, 455) = 27.80, *p* < .001) and GERT scores (*F*(1, 455) = 41.32, *p* < .001 and similar STEM scores (mean: *F*(1, 455) = 1.72, *p* = 0.19, corrected mean: *F*(1, 455) = 0.27, *p* = .60) compared with non-gifted individuals.

Regarding reactivity to emotions, our sample reported similar affect intensity (AIM, *F*(1, 455) = 0.05, *p* = .82) to non-gifted participants. They also reported similar general reactivity to negative emotions (*t*(302) = 0.23, *n.s.*) but less general reactivity to positive emotions (*t*(302) = −3.71, *p* < .001) than the scores provided in Preece et al. (2019). In terms of sensory-processing sensitivity, our sample scored higher than participants in Pluess et al.’s (2020) study, *t*(302) = 5.19, *p* < .001.

Finally, our sample had higher PA (*F*(1, 455) = 6.40, *p* = .01) and similar NA scores (*F*(1, 455) = 0.15, *p* = .70) compared with non-gifted participants. They also reported less perceived stress (*F*(1, 455) = 7.81, *p* = .005) and more satisfaction with life (*F*(1, 455) = 13.05, *p* < .001) than non-gifted individuals.

### 3.2. Analysis of Reaction Times in the DP Task

As the comparison group of non-gifted individuals, we included the sample employed in Nicolet-dit-Félix et al. (2023), which involved 157 participants from the general population. We followed the same analytical procedure as in the original paper to investigate the relationship between EI and attentional bias to emotional stimuli and added the group variable to the analyses.

#### 3.2.1. Data Preprocessing and Analysis

Three participants with less than 80% correct responses in the DP task were excluded from the analyses (one from the Mensa sample and two from the control sample). The analyses were then performed on reaction times (RT) associated with correct responses (3.5% of the responses were incorrect) from 279 participants (124 Mensa members and 155 non-gifted participants). RTs under 200 ms and above 2500 ms were removed (<0.1% of the data), and RTs deviating more than 3sd from each participant’s mean in each condition were then eliminated (1.4% of the data).

As we had a repeated measure experimental design with continuous between-subject explanatory and control variables, we used Linear Mixed-Effects Models (Baayen et al. 2008; Bates et al. 2018) and the statistical software R (R Core Team 2021).

Based on the Box–Cox test’s result (Box and Cox 1964), we inverse transformed RTs. In all models, we used sum coding to define the contrasts of Condition (with −1 for neutral), Gender (with −1 for male), and Group (with −1 for non-gifted participants). We standardized the continuous independent variables around the grand mean.

We started to fit models with the maximal structure that was supported by the data for the random terms for the explanatory variables. When models did not converge or had a singular fit, random terms were removed following the recommendations of Bates et al. (2018).

#### 3.2.2. Replication of Nicolet-dit-Félix et al. (2023) Results Regarding the Role of Emotion Understanding

In Nicolet-dit-Félix et al. (2023), the best model returned significant effects of age, fluid intelligence (Raven matrices), trial number, emotion understanding, and the interaction between emotion understanding and condition. Participants were slower with age but faster with increasing fluid intelligence, trial number, and emotion understanding. Importantly, there was no main condition effect (i.e., RTs were generally not shorter in one of the conditions), but an interaction effect between condition and emotion understanding, showing that when the level of emotion understanding was higher than 1sd from the mean, individuals were faster at identifying letters replacing an emotional compared to a neutral face. Additionally, it was found that the interaction between emotion understanding and condition was better described by a non-linear relationship, including a quadratic term for emotion understanding.

Here, we ran the same analysis as in Nicolet-dit-Félix et al. (2023) and employed Nicolet-dit-Félix et al. sample as the control group to be compared to the Mensa group. We fitted an LME model with inverse RT as the outcome variable, with fixed effects of age, gender^3^, mood, and trial number (since RTs are known to get faster throughout the experiment) as control variables and condition (neutral vs. emotional), emotion understanding, group (non-gifted vs. Mensa) and their interactions as key explanatory variables. We included the quadratic term for emotion understanding in the model. The final model included random intercepts for participants.

The model (see Table 4) revealed significant effects of age (*β* = 1.80, *SE* = 0.18, *p* < .001) and trial number (*β* = −0.30, *SE* = 0.02, *p* < .001) as in Nicolet-dit-Félix et al. (2023) as well as an effect of gender (*β* = 0.36, *SE* = 0.15, *p* = .011), with female participants being slower than male participants in general. Regarding our hypothesis related to hypersensitivity to emotion information, there was no main effect of condition, but an interaction effect between condition and emotion understanding (linear term: *β* = −8.97, *SE* = 4.46, *p* = .044) that was qualified by a group × condition × emotion understanding interaction (linear term: *β* = 9.17, *SE* = 4.46, *p* = .004, quadratic term: *β* = 9.40, *SE* = 3.82, *p* = .01).

**Figure 2:**
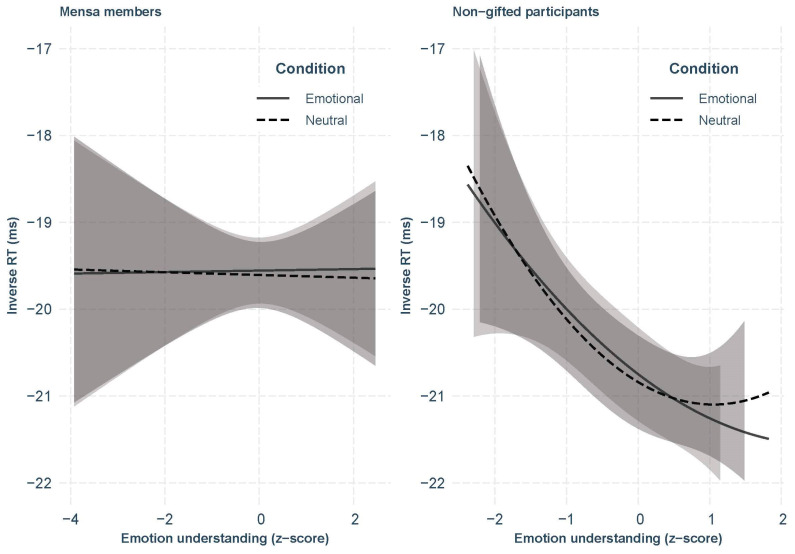
Inverse Reaction Times and 95% CIs as a Function of STEU Score and Condition. *Note.* Gifted individuals’ reaction times did not differ between the conditions nor depending on the level of EI (on the **Left**), whereas non-gifted individuals from Nicolet-dit-Félix et al. (2023) showed shorter reaction times with increasing EI and attentional bias to emotional faces for levels of EI higher than 1sd from the mean (on the **Right**).

In order to interpret the three-way interaction, we split our participants into two groups (i.e., non-gifted and Mensa) and fitted similar models (without the group variable) on each subset. The model fitted on the Mensa sample did show similar effects of age (*β* = 1.94, *SE* = 0.19, *p* < .001) and trial number (*β* = −0.44, *SE* = 0.03, *p* < .001) as in the whole sample, but no main effect of condition nor condition by emotion understanding interaction. In contrast, the model fitted on non-gifted participants showed the effects presented in Nicolet-dit-Félix et al. (2023) (Table 5). In summary, whereas non-gifted participants scoring high on emotion understanding showed an attentional bias toward emotional faces, Mensa members did not show such a bias, even at a high level of emotion understanding (Figure 2).

#### 3.2.3. Exploratory Analyses on the Mensa Sample

To test a potential inhibition or habituation process that could have taken place in Mensa members during the task (i.e., participants getting used to the appearance of emotional faces), we included the block number in interaction with the condition and EI in the model on the Mensa sample. We also modeled reaction times from the first block only, thinking that attentional capture should be maximal at the beginning of the task before diminishing with new stimuli. This did not change the results: all participants were equally fast in both conditions when the block was taken into consideration.

Based on previous findings that negative emotional stimuli capture attention more than positive stimuli (e.g., Pratto and John 1991), we also tested whether participants’ reaction times in the conditions differed depending on the emotional valence of the stimuli. We fitted a similar model, adding emotion (Happiness vs. Anger) in the interaction term. Neither the two-way nor the three-way interactions were significant, suggesting that the emotional valence of the cues did not influence the result.

The model on the Mensa sample was also run either by removing participants who reported Asperger and autism diagnostics or by making subset groups (for depression and anxiety diagnostics). The results remained the same (i.e., no effect of condition or interaction between condition and emotion understanding).

#### 3.2.4. Other Ability EI Facets, Trait EI, and Self-Reported Reactivity to Emotion

The influence of other ability EI facets (i.e., emotion management and emotion recognition) and trait EI were investigated in a similar manner as in the model testing emotion understanding. The models did not show any interaction effect between STEM, GERT, or trait EI and condition and/or group. Finally, we tested whether affect intensity (AIM) or emotional reactivity (PERS) influenced the reaction times in the different conditions. Although AIM predicted an increase in RTs in general, it did not interact with the condition. Emotional reactivity did not have any effect on the model.

## 4. Discussion

The aim of this paper was to describe the characteristics of gifted individuals, in particular, how they perform in emotional intelligence tests and in a task of attention to emotional information. To do so, we first collected descriptive data regarding different aspects of personality and variables that are commonly associated with giftedness, such as hypersensitivity, the prevalence of various types of mental health disorders, satisfaction with life, and the quality of interpersonal relationships.

Our data showed that Mensa members seem to differ from the general population regarding the higher prevalence of Autism Spectrum Disorders (around 1% in Europe, Salari et al. 2022) and ADHD (estimated at about 2.8%, Fayyad et al. 2017). Regarding sensoriy sensitivity, Mensa members seem to be on the higher end of the measure, whereas their scores on affect intensity were similar to those of non-gifted individuals. Finally, we found that Mensa members report less perceived stress and more satisfaction with life than the general population, a finding that supports the harmony hypothesis and the idea that giftedness comes with more capacity to adjust in life.

Data related to the individual characteristics of our participants suggest that Mensa members obtain higher scores on the emotion understanding and emotion recognition facets of EI, but not on emotion management. This corroborates previous findings that IQ is related to ability EI (Ogurlu 2021). Interestingly, the management facet of EI was not higher among Mensa members. This could be explained by the sample tested here, which reported high levels of sensory-processing sensitivity and a higher prevalence of ADHD than the general population. Perhaps stronger sensitivity may make emotion regulation more challenging; in addition, ADHD is associated with difficulties to control impulses, which might also affect emotion management.

Second, to understand how gifted individuals process emotional information, we investigated the EI_P_ component of EI in gifted adults with a DP task allowing us to detect attentional bias toward emotion. As a reminder, EI_P_ corresponds to a fluid component of EI that assesses how individuals experience and respond to emotions (Fiori and Vesely-Maillefer 2018). In a study on the general population, Nicolet-dit-Félix et al. (2023) showed that EI_P_ is related to EI_K_ in individuals with high EI_K_, especially emotion understanding, showing attentional bias toward emotional faces in a DP task. Here, we were interested to see whether gifted participants, who are generally higher on EI_K_, also have specific attention toward emotional information.

We did not find any attentional bias to emotional information in the DP task, nor did we find any association between ability EI and the magnitude of the differences between reaction times in the emotional vs. neutral conditions in Mensa members. In other words, Mensa members were equally fast to respond to probes replacing emotional compared to neutral faces, and their level of EI did not influence this pattern. This suggests that, contrary to findings with the same task in the general population (Nicolet-dit-Félix et al. 2023), EI_P_ seems to be unrelated to EI_K_ in Mensa members. It also suggests that higher knowledge about emotions in Mensa members (as shown in their higher scores on the emotion understanding and emotion recognition facets of ability EI) may not be related to more automatic processing of emotion information.

One might argue that the lack of effect in this study could come from the smaller sample size compared to the one in Nicolet-dit-Félix et al. (2023) or from a smaller range of ability EI scores in Mensa members. While these arguments are admissible with respect to the lack of interaction between EI and condition (emotional vs. neutral), we still think that the main effect of condition should have emerged because our participants had higher ability EI than the general population, meaning that they were similar in this aspect with the participants who showed attentional bias to emotional faces in Nicolet-dit-Félix et al.

Hence, we interpret the lack of the condition effect in Mensa members, even for those high on EI, as reflecting the fact that Mensa members were not particularly responsive to the emotional valence of the stimuli probably because of inhibitory processes, which might have contributed to treating emotional information as purely cognitive. In other words, Mensa members might have been able to suppress the bottom-up processing of emotional content automatically, inhibiting quick capture of attention.

This interpretation is consistent with the signal-suppression hypothesis (Gaspelin and Luck 2019). Trying to reconcile opposite findings in the domain of attentional capture, this hypothesis states that salient items can be suppressed before capturing attention (proactive inhibition), particularly in tasks where the stimuli designed to capture attention are held constant for long blocks of trials, such as in our experiment. This would mean that gifted individuals have more capacity to suppress stimuli that are not directly linked to the task and focus on the task itself (i.e., report a letter) than non-gifted people.

Our results also support findings demonstrating that emotional stimuli may not automatically capture attention, depending on the context in which they are presented, the participants’ goals, and the strategy employed (e.g., Barratt and Bundesen 2012; Glickman and Lamy 2018). Because the task used in this study brought out attentional bias to emotion in a previous study, it is unlikely that the context or the task itself influenced the results in the present study.

We rather argue that the characteristics of the tested Mensa members could explain their lack of attentional bias to emotion in this task. To do this, we refer to the concepts of people- and thing-centered intelligence (Bryan and Mayer 2020; Mayer 2008), which are based on the type of content (i.e., people or things) individuals reason about. Among thing-centered intelligences, we find quantitative knowledge about numbers or visuospatial abilities and emotional or social intelligences among people-centered ones. In a recent meta-analysis, Bryan and Mayer (2020) demonstrated that people-centered intelligences, which include EI, are more highly correlated among themselves than they are with thing-centered intelligences. The pattern was similar for thing-centered intelligences. This meta-analysis also confirmed that EI and intelligence are to some extent related, but that this relationship is moderate: Correlation was 0.29 between EI and fluid intelligence and 0.35 between EI and comprehension knowledge. All in all, these findings suggest that Mensa members might have a higher thing-centered intelligence than people-centered intelligence and that the association with people-centered intelligence might be stronger for EI_K_ than EI_P_.

### Strengths and Limitations

It is important to note that his study was conducted on a specific subset of the whole gifted population, namely Mensa members. For this reason, the results presented here might not be generalized to the whole gifted population. It is indeed possible that Mensa members present some shared characteristics that are not found in all gifted individuals. Mensa members are indeed those gifted individuals that decided to take part in an association especially conceived for high-IQ individuals. They must have their IQ evaluated in order to do so, and the decision to take such a test might be explained by different reasons, such as difficulties in their social or personal life and the need for belongingness, which could be resolved by meeting individuals with similar characteristics.

As participation was voluntary, we cannot exclude that people who decided to take part in this study already had some sort of interest in emotional intelligence (either because they felt they were good at it or, on the opposite, because they felt as if they could learn from the study).

Further research with a larger sample of gifted individuals and with a more similar control group is also needed to better understand their characteristics regarding emotional capacities.

Despite these limitations, our study provides additional data on gifted adults, which is a valuable addition to the literature. Data on gifted adults are scarce because, in addition to representing only 2% of the population, gifted adults are hard to detect. Gifted individuals that were not identified at school have few chances of being identified once they are adults, and not all gifted adults know they are gifted (Dijkstra et al. 2012).

## 5. Conclusions

In this study, we collected information on different aspects of emotional competence in gifted individuals from Mensa society. More specifically, we tested how Mensa members perform on emotional intelligence tasks in comparison with non-gifted individuals and whether they show an attentional bias toward emotional stimuli. Whereas Mensa members obtained higher scores on several measures of EI, they did not show any attentional bias toward emotional expressions as individuals from the general population did.

This study offers new insights into gifted individuals’ emotional competence and provides new information on the way Mensa members treat emotional stimuli. It supports previous findings showing that highly intelligent individuals perform better on performance-based tests evaluating EI. In addition, it compares EI_P_ in gifted and non-gifted individuals and suggests that higher emotional knowledge in gifted individuals is not related to higher emotional information processing, contrary to the general population. This finding confirms the utility of having a theory that distinguishes two EI components, an emotion knowledge component or EI_K_ and emotion–information processing or EI_P_ component, which may capture different aspects of EI: one more related to what people know about emotions and the other to how people respond to and treat emotional information. Whereas Mensa members seem to have wide knowledge about emotions, they do not seem to possess any preferential treatment of emotional information. Further research might ascertain whether different scores on the two EI components are associated with different behavioral tendencies in social and emotional situations.

In summary, the results of this study suggest that Mensa participants are not particularly responsive to the emotional valence of stimuli; this might be related to the role of inhibitory processes (a factor correlated with intelligence), which might have contributed to treating emotional information as purely cognitive. Additional research is needed to specifically test this possibility.

## Figures and Tables

**Table 1:** Education and Living Area of the Mensa Sample in the First Session of the Study (N = 304).

Education	Frequency	Percentage
Compulsory education	6	2.0
Apprenticeship	8	2.6
Upper secondary level	46	15.1
Bachelor’s Degree	88	28.9
Master’s Degree	108	35.5
PhD or higher	27	8.9
Other	21	6.9
**Living Area**		
Urban	112	36.8
Suburban	107	35.2
Rural	81	26.6
Remote	4	1.3

**Table 2:** Descriptive Statistics (Means and Standard Deviations) for the Variables in the First Session of the Study for Mensa members (N = 303) and Controls from Nicolet-dit-Félix et al. (2023, N = 155) and Correlations between the Variables for the Mensa Sample.

	Controls	Mensa	1	2	3	4	5	6	7	8	9	10	11
1. Age	28.9 (9.8)	49.2 (15.6)											
2. STEU	0.62 (0.12)	0.74 (0.11)	−.08										
3. STEM	0.60 (0.15)	0.63 (0.10)	.11	.29 ***									
4. GERT	0.58 (0.15)	0.62 (0.11)	−.31 ***	.00	.05								
5. AIM	75.6 (11.5)	71.3 (13.1)	−.12 *	−.12 *	.00	.18 **							
6. PANAS p	31.5 (7.8)	33.5 (7.1)	−.02	.00	−.04	−.02	.24 ***						
7. PANAS n	25.0 (7.8)	21.8 (7.6)	−.35 ***	−.02	−.11	.17 **	.28 ***	−.10					
8. PERS p		31.6 (7.5)	.16 **	.11	.14 *	−.13 *	.29 ***	.43 ***	−.17 **				
9. PERS n		26.6 (8.4)	−.19 **	.10	.02	.03	.38 ***	−.15 *	.52 ***	−.01			
10. HSPS		4.62 (0.94)	−.17 **	−.13 *	−.05	.24 ***	.47 ***	.01	.42 ***	.03	.48 ***		
11. PSS	20.6 (6.8)	15.7 (6.8)	−.22 ***	−.02	−.05	.17 **	.28 ***	−.34 ***	.66 ***	−.24 ***	.56 ***	.43 ***	
12. SWLS	20.6 (7.2)	22.2 (6.8)	.00	.08	.05	−.04	.08	.46 ***	−.24 ***	.31 ***	−.24 ***	−.08	−.51 ***

Note: STEU: Situational Test of Emotion Understanding, STEM: Situational Test of Emotion Management, GERT: Geneva Emotion Recognition Test, AIM: Affect Intensity Measure, PANAS: Positive and Negative Affect Schedule, PERS: Perth Emotional Reactivity Scale, HSPS: Highly Sensitive Person Scale, PSS: Perceived Stress Scale, SWLS: Satisfaction with Life Scale. PERS and HSP were not measured in Nicolet-dit-Félix et al. (2023). * *p* < .05, ** *p* < .01, *** *p* < .001.

**Table 3:** Percentages of Participants Reporting Symptoms or Being Diagnosed with different Syndromes in the First Session of the Study (N = 304).

	Anxiety	Bipolar	Depression	Autism	Asperger	ADHD	Borderline	Other
Symptoms	18.8	2.3	10.9	10.9	11.8	7.9	1.3	3.6
Diagnosis	7.9	1.0	12.5	2.6	3.9	4.9	0.7	5.6

**Table 4:** Models testing the interaction effect between group (non-gifted vs. Mensa), emotion understanding (STEU), and condition (neutral vs. emotional) on reaction times in the DP task, controlling for age, trial number, gender, and mood. The dependent variable is inverse reaction time.

Predictors	Estimates	CI	*p*
Intercept	−20.4	−20.75–−20.06	<0.001
Condition (1 = emotional)	−0.02	−0.06–0.02	0.393
Group (1 = Mensa)	−0.36	−0.76–0.04	0.074
Age	1.77	1.41–2.13	<0.001
Trial Number	−0.3	−0.33–−0.26	<0.001
Gender (1 = female)	0.36	0.06–0.65	0.018
Mood	0.19	−0.10–0.48	0.207
Condition x Group	0.04	−0.00–0.08	0.056
STEU (linear)	−49.22	−119.10–20.66	0.167
STEU (quadratic)	21.58	−38.62–81.77	0.482
Condition × STEU (linear)	−8.97	−17.70–−0.23	0.044
Condition × STEU (quadratic)	−5.67	−13.17–1.82	0.138
Group × STEU (linear)	60.72	−9.88–131.33	0.092
Group × STEU (quadratic)	−22.9	−83.32–37.52	0.458
Group × Condition × STEU (linear)	9.17	0.44–17.91	0.04
Group × Condition × STEU (quadratic)	9.4	1.90–16.89	0.014
**Random Effects**			
σ^2^	10.77		
τ^2^	5.54 _PARTICIPANT_		
ICC	0.34		
N	276 _PARTICIPANT_		
Observations	33,558		
Marginal R^2^/Conditional R^2^	0.155/0.442		

**Table 5:** Model testing in each sample (Mensa and non-gifted participants) the effects of condition (neutral vs. emotional) and emotion understanding on reaction times in the DP task, controlling for age, trial number, gender, and mood. The dependent variable is inverse reaction time.

Predictors	Non-Gifted Participants			Mensa Members		
	Estimates	CI	*p*	Estimates	CI	*p*
Intercept	−20.74	−21.17–−20.31	<0.001	−19.61	−19.98–−19.24	<0.001
Condition (1 = emotional)	−0.01	−0.06–0.04	0.69	0.03	−0.02–0.07	0.289
STEU (linear)	−89.92	−146.38–−33.46	0.002	6.65	−39.66–52.96	0.778
STEU (quadratic)	27.84	−27.19–82.88	0.321	3.63	−43.63–50.89	0.88
Age	0.9	0.49–1.31	<0.001	1.92	1.54–2.31	<0.001
Trial Number	−0.22	−0.27–−0.17	<0.001	−0.39	−0.44–−0.35	<0.001
Gender (1 = female)	0.37	−0.07–0.82	0.096	0.38	−0.01–0.76	0.054
Mood	0.38	−0.03–0.80	0.072	−0.07	−0.45–0.31	0.728
Condition × STEU (linear)	−7.5	−14.46–−0.53	0.035	1.5	−4.21–7.22	0.606
Condition × STEU (quadratic)	−9.09	−16.05–−2.12	0.011	2.29	−3.43–8.01	0.432
**Random Effects**						
σ^2^	12.62			8.5		
τ^2^	6.44 _PARTICIPANT_			4.38 _PARTICIPANT_		
ICC	0.34			0.34		
N	153 _PARTICIPANT_			123 _PARTICIPANT_		
Observations	18,416			15,142		
Marginal R^2^/Conditional R^2^	0.089/0.397			0.235/0.495		

## Data Availability

The data presented in this study are openly available on OSF at https://osf.io/7zxhd/.

## Notes

1 The 2% threshold corresponds to a score of 131 for the Wechsler tests (WISC and WAIS), 132 for the Stanford-Binet test, or 150 for the Cattell test.

2 One individual, who scored very low on STEU and STEM measures, was removed from the analysis.

3 Gender was not a significant predictor in Nicolet-dit-Félix et al. (2023) but was still included in this analysis since it is known to be related to reaction times. Three participants who indicated “other” for this variable were removed from the analysis. Fluid intelligence was not included as we did not ask the Mensa sample to complete Raven’s matrices.
